# Association of Single Measurement of dipstick proteinuria with physical performance of military males: the CHIEF study

**DOI:** 10.1186/s12882-020-01948-w

**Published:** 2020-07-18

**Authors:** Chia-Hao Fan, Ssu-Chin Lin, Kun-Zhe Tsai, Tsung-Jui Wu, Yen-Po Lin, Yu-Kai Lin, Shao-Chi Lu, Chih-Lu Han, Gen-Min Lin

**Affiliations:** 1 Department of Nursing, Hualien Armed Forces General Hospital, Hualien, Taiwan; 2 Department of Medicine, Hualien Armed Forces General Hospital, No. 163, Jiali Rd., Xincheng Township, Hualien, 97144 Taiwan; 3 Department of Critical Care Medicine, Taipei Tzu Chi Hospital, New Taipei, Taiwan; 4 Department of Medicine, Tri-Service General Hospital, National Defense Medical Center, Taipei, Taiwan; 5 Department of Medicine, Taipei Veterans General Hospital, Taipei, Taiwan; 6 Department of Preventive Medicine, Northwestern University Feinberg School of Medicine, Chicago, IL 60611 USA

**Keywords:** Proteinuria, Physical performance, Military male adults

## Abstract

**Background:**

Proteinuria, a marker of kidney injury, may be related to skeletal muscle loss. Whether the severity of proteinuria is associated with physical performance is unclear.

**Methods:**

We examined the association of proteinuria severity with physical performance cross-sectionally in 3357 military young males, free of chronic kidney disease, from the cardiorespiratory fitness and hospitalization events in armed Forces (CHIEF) study in Taiwan. The grades of proteinuria were classified according to one dipstick urinalysis which were collected at morning after an 8-h fast as unremarkable (0, +/−, and 1+), moderate (2+) and severe (3+ and 4+). Aerobic physical performance was evaluated by time for a 3000-m run and anaerobic physical performance was evaluated by numbers of 2-min sit-ups and 2-min push-ups, separately. Multiple linear regressions were used to determine the relationship.

**Results:**

As compared with unremarkable proteinuria, moderate and severe proteinuria were dose-dependently correlated with 3000-m running time (β: 4.74 (95% confidence intervals (CI): − 0.55, 10.02) and 7.63 (95% CI: 3.21, 12.05), respectively), and inversely with numbers of 2-min push-ups (β = − 1.13 (− 1.97, − 0.29), and − 1.00 (− 1.71, − 0.28), respectively) with adjustments for age, service specialty, body mass index, blood pressure, alcohol intake, smoking, fasting plasma glucose, blood urea nitrogen, serum creatinine and physical activity. However, there was no association between proteinuria severity and 2-min sit-ups.

**Conclusions:**

Our findings show a relationship of dipstick proteinuria with aerobic physical performance and parts of anaerobic physical performance in military healthy males. This mechanism is not fully understood and requires further investigations.

## Background

Protein is one of the major materials such as the actin and myosin components of sarcomere constituting skeletal muscle fibers in the body [1]. Under normal conditions, protein loss is less than 150 mg per day and excreted by the kidney in healthy adults [2]. Proteinuria is defined as excess proteins excreted in the urine and commonly observed in several clinical situations [3]. Pathological proteinuria is related to urinary tract abnormalities (e.g. hematuria), systemic inflammation, diabetes, hypertension, malignancies, dehydration, drugs and chronic kidney disease [4–6]. By contrast, physiological proteinuria is usually asymptomatic and associated with intense physical activity, also referred as exercise-induced proteinuria [7, 8].

The pathophysiological mechanisms of proteinuria can be reasoned by an increase of glomerular capillary permeability to proteins, reduced protein reabsorption in the renal tubules, and overflow of low-molecular-weight proteins [9]. With regard to exercise-induced proteinuria, the main cause is not clarified, but the renin-angiotensin system and prostaglandins are considered to play a major role [10]. The plasma angiotensin II concentration increases at exercise, possibly resulting in filtration of proteins through the glomerular membrane [11]. Strenuous exercises which activate the sympathetic nervous system, secrete catecholamine and produce lactate, would increase the permeability of glomerular capillary membrane and related excessive protein excretions as well [12]. In addition, intense physical activity is at higher risk of rhabdomyolysis and dehydration, and leads to presence of proteinuria [13]. Previous studies have shown that exercise-induced proteinuria is associated with the amount and the length of exercise performed [14]. In an elderly cohort higher albuminuria is a predictor of a decline of physical performance > 2 years [15]. It is important to monitor proteinuria in the general population; however in young adults, the relationship with exercise capacity has not been explored.

Military personnel are a special group of young individuals, required to receive intensive training regularly. We hypothesized that presence of proteinuria in military personnel may be related to skeletal muscle injury caused by rigorous training and thus they have poor exercise performance. In this study, we aimed to investigate the association of proteinuria severity with physical performance in a military population in Taiwan.

## Methods

### Study population

The study of cardiorespiratory fitness and hospitalization events in armed forces (CHIEF) was performed in eastern Taiwan [16–20]. All participants carried out a formal health examination and self-reported a questionnaire for their habits of cigarette smoking and alcohol intake status (current vs. former or never) and physical activity in leisure times per week in the past 6 months in Hualien Armed Forces General Hospital. After the health examination, all participants took at least one of the three exercise tests including 2-min sit-ups, 2-mininute push-ups, and a 3000-m run at the Hualien Military Physical Training and Testing Center. The study design of CHIEF study has been described in detail previously [19, 21–25]. In 2014, there were 4080 participants receiving the health examination and exercise tests in the study. We excluded 312 males for the BUN > 20 mg/dL or serum creatinine > 1.20 mg/dL, and presence of sediments, casts, crystals, glucose, bacteria, positive nitrates, leukocytes and red blood cells in the urine, and the urine specific gravity > 1.020 or < 1.010, leaving a final sample of 3357 males for analysis. In addition, the remaining sample of 411 females were used for a sensitivity test.

### Anthropometric and blood tests

Before taking the laboratory examinations, each participant took his temporal temperature and was interviewed by a military physician. If there were any infection symptoms or signs along with the body temperature over 37^。^C, the examinee would be asked to make another appointment while recovery from the sickness to complete the health examination. Anthropometric parameters of body height and body weight were assessed in a standing position of each participant. Body mass index was defined as a ratio of body weight (kilogram) divided by square of height (meter^2^). Waist circumference was measured at the midpoint between the highest point of the iliac crest and the last palpable rib. All participants measured their blood pressure once over right upper arm in a sitting position following a rest for at least 15 min by an automated blood pressure monitor (FT-201, Parama-Tech Co Ltd., Fukuoka, Japan). Laboratory data of blood biochemical tests for blood urea nitrogen (BUN), serum creatinine and fasting plasma glucose were obtained in the morning after a 12-h fast.

### Dipstick urine examination

To avoid a stress status of participants, possibly confounding the interpretation of proteinuria, the urine sample was collected prior to a venous puncture for drawing the blood sample in the morning. Urine proteins were measured using the AUTION dipsticks (ARKRAY. Inc., Shiga, Japan) by a fully automated urine chemistry analyzer (AX-4030, ARKRAY. Inc., Shiga, Japan). On the basis of the clinical classifications recommended by the National Kidney Foundation [26], the concentrations of urine proteins of 30–99 mg/dL are defined as “1+”, 100–299 mg/dL as “2+”, 300–999 mg/dL as “3+” and ≥ 1000 mg/dL are defined as “4+”. The intra-assay coefficient of variation in grading proteinuria was 6.08%.

### Physical performance assessment

Aerobic physical performance was evaluated by 3000-m running time, a strong indicator of the velocity at lactate (anaerobic) threshold [27]. All runs were carried out on a flat playground at 4:00 PM only if the risk coefficient of heat stroke, the product of outdoor temperature (°C) and relative humidity (%) × 0.1, was lower than 40 and no raining. Anaerobic physical performance was separately evaluated by numbers of 2-min push-ups and 2-min sit-ups. The procedures were standardized performing on a sponge pad and scored by computer infrared sensing systems. All testing courses of each participant were monitored by the observing officers and video recorded. Since the exercise tests are closely related to the rank promotion and military award, the performance is regarded as the best physical fitness of each participant. This study was reviewed and approved by the Institutional Review Board of the Mennonite Christian Hospital (No. 16–05-008) in Hualien of Taiwan and written informed consent was obtained from all participants.

### Statistical analysis

The severity of proteinuria were classified to 3 groups based on the results of dipsticks as unremarkable (grades: 0, +/−, 1+, *N* = 2011), moderate (grades: 2+, *N* = 1058) and severe (grades: 3+ and 4+, *N* = 288). The characteristics of subjects were expressed as mean ± standard deviation (SD) for continuous data, and numbers with percentages for categorical data. The relationship of moderate and severe proteinuria with the performance in each exercise (i.e., time for a 3000-m run, numbers of 2-min push-ups and numbers of 2-min sit-ups) were investigated by using analysis of covariance (ANCOVA), and the results were expressed as mean ± standard error (SE). Multiple stepwise linear regression analyses of each exercise performance with moderate and severe proteinuria, relative to unremarkable proteinuria, were also performed as the main analysis. Since the performance in each exercise was centrally distributed (supplemental Figure), it might not be appropriate using the linear regression analysis to evaluate the possibility of remarkable proteinuria for the highest or lowest physical performance. Therefore, multiple logistic regression analyses were used to determine the odds ratio (OR) of being the superior (highest 10th percentile) and the inferior (lowest 10th percentile) performers in each exercise with moderate and severe proteinuria compared to unremarkable proteinuria (secondary analysis). The levels for the superior/inferior performances in each exercise were alternatively set as 5 and 16% for a comparison with 10%.

In model 1, age and military service specialty were adjusted. In model 2, body mass index, BUN and serum creatinine were additionally adjusted. In model 3, systolic blood pressure, fasting plasma glucose, smoking status, alcohol intake status and physical activity were further adjusted. These potential confounders for the models were chosen based on prior published associations with physical performance [28] and those with a significant difference among the groups. A value of *p* < 0.05 was considered significant. Statistical analyses were done with a standard program (Statistical Package for Social Sciences, SPSS, version 25.0).

## Results

### Subject characteristics

The subject characteristics of each group are revealed in Table 1. The males with unremarkable proteinuria had relatively older ages, greater body mass index and waist circumference, a lower prevalence of current cigarette smokers, and lower concentrations of BUN, serum creatinine and fasting plasma glucose than those with moderate and severe proteinuria. On the contrary, the males with severe proteinuria had a higher level of systolic blood pressure and higher concentrations of BUN, serum creatinine and fasting plasma glucose than those with unremarkable or moderate proteinuria.

**Table 1:** Baseline Characteristics of the Study Cohort (*n* = 3357)

Characteristics	Unremarkable Proteinuria (*n* = 2011)	Moderate Proteinuria (*n* = 1058)	Severe Proteinuria (*n* = 288)	*p*-value
Age (years)	29.80 ± 5.60	28.92 ± 6.13	27.89 ± 5.99	< 0.01
Specialty, n (%)				
Army	989 [49.2]	547 [51.7]	146 [50.7]	0.03
Navy	474 [23.6]	197 [18.6]	63 [21.9]	
Air force	548 [27.2]	314 [29.7]	79 [27.4]	
Body mass index (kg/m^2^)	25.04 ± 3.00	24.63 ± 3.19	24.80 ± 3.21	< 0.01
Waist circumference (cm)	83.83 ± 7.62	82.73 ± 8.34	83.16 ± 8.40	< 0.01
Systolic blood pressure (mmHg)	118.68 ± 12.84	117.64 ± 13.25	119.68 ± 12.85	0.02
Diastolic blood pressure (mmHg)	70.80 ± 9.93	70.22 ± 10.35	70.47 ± 10.45	0.31
Blood test				
Blood urea nitrogen, BUN (mg/dL)	12.66 ± 2.75	13.25 ± 2.90	13.43 ± 3.17	< 0.01
Serum creatinine (mg/dL)	0.96 ± 0.11	0.97 ± 0.11	0.99 ± 0.12	< 0.01
Fasting plasma glucose (mg/dL)	93.19 ± 12.57	93.43 ± 13.51	96.37 ± 18.93	< 0.01
Alcohol consumption, n (%)				
Former or never alcohol intake	1151 [57.2]	567 [53.6]	164 [59.6]	0.14
Current alcohol intake	860 [42.8]	491 [46.4]	124 [43.1]	
Betel nut chewing, n (%)				
Former or never chewer	1761 [89.0]	912 [87.2]	250 [88.3]	0.34
Current chewer	218 [11.0]	134 [12.8]	33 [11.7]	
Cigarette smoking, n (%)				
Former or never smoker	1285 [64.9]	613 [58.6]	165 [58.3]	< 0.01
Current smoker	694 [35.1]	433 [41.4]	118 [41.7]	
Physical activity, n (%)				
Never or occasionally	404 [20.1]	235 [22.2]	66 [22.9]	0.38
1–2 times/week	773 [38.4]	386 [36.5]	97 [33.7]	
≥ 3 times/week	834 [41.5]	437 [41.3]	125 [43.4]	

Continuous variables are expressed as mean ± standard deviation (SD), and categorical variables as n [%]. Unremarkable proteinuria was defined as dipstick proteinuria grades: 0, +/−, or 1+; moderate proteinuria as grades: 2+; and severe proteinuria as grades: 3+ or 4+

### Group means comparisons

Table 2 shows the number of military males specifically for an exercise test and the mean of physical performance in the three proteinuria groups. There were significant differences in 3000-m running time (857.2 s vs. 861.5 s vs. 873.0 s, *p* < 0.01) and 2-min push-ups numbers (49.6 vs. 48.5 vs. 47.5, p < 0.01) among the unremarkable proteinuria, moderate proteinuria, and severe proteinuria groups after adjusting for all covariates in model 3. However, there were similar 2-min sit-ups numbers among the three proteinuria groups in models 1–3. In addition, since there was only one young female categorized in the moderate proteinuria group, the moderate and severe proteinuria groups were combined to the remarkable proteinuria group in the sensitivity analysis which demonstrated similar tendencies for each physical performance, despite statistically insignificance (supplemental Table 1).

**Table 2:** Differences in Each Exercise Performance

	2-min push-ups (numbers)			2-min sit-ups (numbers)			3000-m run (seconds)		
	N	Mean (SE)	*p*-value	n	mean (SE)	*p*-value	n	mean (SE)	*p*-value
Model 1									
Unremarkable proteinuria	1996	49.5 (0.2)	0.01^1^	2003	47.7 (0.2)	0.35^1^	1816	857.5 (1.7)	< 0.01^1^
Moderate proteinuria	1047	48.7 (0.4)	0.06^2^	1050	47.3 (0.2)	0.20^2^	944	860.3 (2.3)	0.31^2^
Severe proteinuria	288	47.6 (0.7)	0.01^3^	288	47.2 (0.5)	0.33^3^	262	873.8 (4.4)	< 0.01^3^
Model 2									
Unremarkable proteinuria	1988	49.7 (0.3)	< 0.01^1^	1996	47.8 (0.2)	0.18^1^	1810	856.8 (1.6)	< 0.01^1^
Moderate proteinuria	1047	48.4 (0.4)	< 0.01^2^	1050	47.2 (0.2)	0.10^2^	944	862.2 (2.3)	0.07^2^
Severe proteinuria	288	47.3 (0.7)	< 0.01^3^	288	47.0 (0.5)	0.17^3^	262	874.1 (4.3)	< 0.01^3^
Model 3									
Unremarkable proteinuria	1964	49.6 (0.3)	< 0.01^1^	1972	47.7 (0.2)	0.36^1^	1778	857.2 (1.6)	< 0.01^1^
Moderate proteinuria	1035	48.5 (0.4)	0.01^2^	1038	47.3 (0.2)	0.18^2^	932	861.5 (2.2)	0.11^2^
Severe proteinuria	282	47.5 (0.7)	< 0.01^3^	282	47.2 (0.5)	0.29^3^	256	873.0 (4.2)	< 0.01^3^

^1^Overall *p*-value; ^2^Unremarkable proteinuria vs moderate proteinuria; ^3^Unremarkable proteinuria vs moderate proteinuria

Mean ± standard error (SE) for each exercise performance estimated using analysis of covariance with adjustments for Model 1: age and service specialty adjustments; Model 2: the covariates in Model 1, body mass index, BUN and serum creatinine adjustments; Model 3: the covariates in Model 2, systolic blood pressure, fasting plasma glucose, alcohol intake status, cigarette smoking status, and weekly physical activity adjustments

### Multiple linear regressions

The results of multiple linear regressions of each exercise performance, with moderate and severe proteinuria relative to unremarkable proteinuria, shown in Table 3 are consistent with the findings of ANCOVA in Table 2. As compared with unremarkable proteinuria, moderate proteinuria and severe proteinuria were dose-dependently and positively correlated with time for a 3000-m run (β =4.74 and 7.63; *p*-values =0.07 and < 0.01, respectively) and inversely with 2-min push-ups numbers (β = − 1.13 and − 1.00; both p-values < 0.01, respectively) in model 3. However, moderate proteinuria and severe proteinuria were not correlated with 2-min sit-ups numbers in models 1–3. The sensitivity test for females also showed similar tendencies in each physical performance, despite insignificance in models 1–3 (supplemental Table 2).

**Table 3:** Multiple Liner Regressions of Dipstick Proteinuria Severity With Each Exercise Performance

	Moderate proteinuria			Severe proteinuria		
	β-value	95% CI	*p*-value	β-value	95% CI	*p*-value
Model 1						
2-min push-ups (n)	−0.81	−1.67 – 0.048	0.06	−1.01	−1.74 – − 0.28	< 0.01
2-min sit-ups (n)	−0.37	− 0.97 – 0.23	0.22	− 0.26	− 0.77 – 0.24	0.30
3000-m run (sec)	3.18	−2.33 – 8.70	0.25	8.45	3.81–13.08	< 0.01
Model 2						
2-min push-ups (n)	−1.26	− 2.11 – −0.41	< 0.01	− 1.20	− 1.92 – − 0.48	< 0.01
2-min sit-ups (n)	− 0.59	−1.19 – 0.01	0.05	− 0.41	−0.92 – 0.091	0.10
3000-m run (sec)	5.95	0.57–11.33	0.03	8.83	4.32–13.35	< 0.01
Model 3						
2-min push-ups (n)	−1.13	−1.97 – −0.29	< 0.01	− 1.00	−1.71 – − 0.28	< 0.01
2-min sit-ups (n)	− 0.47	− 1.06 – 0.12	0.12	− 0.30	−0.79 – 0.20	0.24
3000-m run (sec)	4.74	−0.55 – 10.02	0.07	7.63	3.21–12.05	< 0.01

Data are presented as βand 95% confidence intervals (CI) using Pearson’s correlation coefficient for Model 1: age and service specialty adjustments; Model 2: the covariates in Model 1, body mass index, blood urea nitrogen and serum creatinine adjustments; Model 3: the covariates in Model 2, systolic blood pressure, fasting plasma glucose, alcohol intake status, cigarette smoking status, and weekly physical activity adjustments

### Multiple logistic regressions

The results of multiple logistic regressions of the best 10% and the worst 10% performances in each exercise, with moderate and severe proteinuria relative to unremarkable proteinuria, are shown in Table 4. As compared to those with unremarkable proteinuria, the males with moderate proteinuria and the males with severe proteinuria were more likely to be the worst 10% performers in the 3000-m run test in models 3 (OR = 1.30 and 1.93, respectively). In addition, the males with severe proteinuria but not the males with moderate proteinuria were more likely to be the worst 10% performers in the 2-min push-ups test (OR: 1.77). On the contrary, the males with moderate proteinuria and the males with severe proteinuria were not associated with the performances in 2-min sit-ups in models 1–3. The results of multiple logistic regressions of the best and the worst performances in each exercise at the level of 5 and 16%, respectively with moderate and severe proteinuria relative to unremarkable proteinuria, are in line with that at the level of 10%, which are shown in supplemental Table 3.

**Table 4:** Multiple Logistic Regressions of Dipstick Proteinuria Severity With the Best 10% and the Worst 10% of Each Exercise Performance

	Moderate proteinuria			Severe proteinuria			Unremarkable proteinuria
	OR	95% CI	*p*-value	OR	95% CI	*p*-value	Ref
Top 10% of performance level							
Model 1							
2-min push-ups ≥ 60 numbers	0.96	0.76–1.21	0.71	1.20	0.70–1.50	0.90	1.00
2-min sit-ups ≥ 59 numbers	0.83	0.64–1.07	0.14	0.82	0.54–1.25	0.36	1.00
3000-m run ≤783 s	1.03	0.85–1.25	0.75	0.92	0.66–1.27	0.60	1.00
Model 2							
2-min push-ups ≥ 60 numbers	0.90	0.71–1.14	0.39	0.98	0.67–1.43	0.90	1.00
2-min sit-ups ≥ 59 numbers	0.79	0.61–1.02	0.07	0.77	0.50–1.18	0.22	1.00
3000-m run ≤783 s	1.04	0.86–1.26	0.70	0.92	0.67–1.28	0.62	1.00
Model 3							
2-min push-ups ≥ 60 numbers	0.94	0.74–1.19	0.58	1.03	0.69–1.52	0.90	1.00
2-min sit-ups ≥ 59 numbers	0.82	0.63–1.06	0.12	0.78	0.50–1.21	0.26	1.00
3000-m run ≤783 s	1.04	0.86–1.26	0.66	0.90	0.65–1.25	0.52	1.00
Bottom 10% of performance level							
Model 1							
2-min push-ups ≤ 37 numbers	1.08	0.85–1.38	0.53	1.70	1.20–2.42	< 0.01	1.00
2-min sit-ups ≤ 40 numbers	1.24	0.96–1.60	0.10	0.87	0.52–1.45	0.59	1.00
3000-m run ≥934 s	1.25	0.96–1.62	0.09	2.01	1.39–2.92	< 0.01	1.00
Model 2							
2-min push-ups ≤ 37 numbers	1.16	0.91–1.49	0.23	1.85	1.29–2.69	< 0.01	1.00
2-min sit-ups ≤ 40 numbers	1.26	0.97–1.63	0.08	0.91	0.55–1.53	0.72	1.00
3000-m run ≥934 s	1.31	1.00–1.71	0.04	2.05	1.40–3.01	< 0.01	1.00
Model 3							
2-min push-ups ≤ 37 numbers	1.16	0.90–1.49	0.25	1.77	1.23–2.56	< 0.01	1.00
2-min sit-ups ≤ 40 numbers	1.21	0.93–1.57	0.16	0.83	0.49–1.41	0.48	1.00
3000-m run ≥934 s	1.30	0.99–1.69	0.05	1.93	1.31–2.84	< 0.01	1.00

Data are presented as odds ratios (OR) and 95% confidence intervals (CI) using multiple logistic regression analysis for Model 1: age and service specialty adjustments; Model 2: the covariates in Model 1, body mass index, blood urea nitrogen and creatinine adjustments; Model 3: the covariates in Model 2, systolic blood pressure, fasting plasma glucose, alcohol intake status, cigarette smoking status and weekly exercise frequency adjustments

## Discussion

Our principal findings were that more severe proteinuria in one urine dipstick test was associated with lower cardiorespiratory (aerobic) and part of anaerobic physical performance, particularly for muscular endurance of the chest wall and upper extremities in physically fit male adults with euvolemia and without chronic kidney disease, independent of age, body mass index, kidney function, fasting plasma glucose, cigarette smoking, alcohol intake and physical activity. While the effect sizes may be small, the results are still concerning given the young age and general good health of the study population, especially because proteinuria is suspected to worsen over time.

Previous studies have revealed that there was an association between muscle wasting and a decrease of renal function. People with chronic kidney disease are predisposed to muscle wasting and vice versa, those with sarcopenia have a decrease of glomerular filtration rate, which may progress to chronic kidney disease status and result in presence of persistent proteinuria [29–32]. Our findings were in line with this concept by the characteristics of lower body weight and greater BUN and serum creatinine levels which were mainly found in the males with moderate or severe proteinuria. In addition, it is reasonable that decreased muscle mass in the study subjects could lead to lower aerobic physical performance and impair part of anaerobic physical performance.

The other important findings were that higher prevalence of higher fasting plasma glucose levels and cigarette smoking were observed in those with moderate or severe proteinuria. As is known, exercise induced proteinuria has been associated with higher insulin resistance [33] which in turn may lead to muscular protein degradations by suppression of Phosphatidylinositol 3-kinase (PI3K)/protein kinase B (Akt) signaling and subsequently activation of caspase-3 and the ubiquitin-proteasome proteolytic pathway [34]. In addition, current cigarette smoking can increase inflammation [35] and induce insulin resistance [36], which may lead to muscle wasting, and has been associated with the emergence of dipstick proteinuria in healthy middle aged populations [37, 38]. Therefore, insulin resistance and cigarette smoking may be a mediator for the relationship of proteinuria with lower physical performance.

Our study had several advantages. First, the laboratory examinations and the procedures of exercise tests were all performed standardly. Second, a large number of military males were included for the analysis which could provide sufficient power detecting the difference between the groups. Third, 91.5% of the male participants were retained in the analysis that the selection bias would be minimized. Fourth, the daily schedule of military such as training program was unified so that the bias from unmeasured confounders could be controlled at baseline. However, there were several weak points in this study. First, the presence and grades of proteinuria were measured only by one urine dipstick test, which was easily influenced by many physiological and environmental conditions [39]. Second, although numerous covariates were adjusted, we could not completely avoid the existence of other potential confounders that may lead to a bias. Third, although the statistical differences in the outcome of interests (physical performance) were significant, probably because of the large sample size in this study, the absolute differences were relatively small, which might limit the practical applications.

## Conclusion

Our findings show a dose-dependent relationship of dipstick proteinuria with aerobic physical performance and part of anaerobic physical performance in military healthy males free of chronic kidney disease. Cigarette smoking and increased insulin resistance may play a potential role on the relationship between remarkable proteinuria and muscle wasting, leading to impaired physical performance. This mechanism is not fully understood and requires further investigations.

## Supplementary information

**Additional file 1.**

**Additional file 2.**

## Data Availability

As the study materials were obtained from the military in Taiwan, the data were confidential and not allowed to be opened in public. If there are any needs for clarification, the readers can contact Colonel Dr. Gen-Min Lin, the corresponding author for sharing the data.

## Abbreviations

Akt: Protein kinase B
ANCOVA: Analysis of covariance
BUN: Blood urea nitrogen
CHIEF: Cardiorespiratory fitness and hospitalization events in armed forces
CI: Confidence interval
OR: Odds ratio
PI3K: Phosphatidylinositol 3-kinase
SD: Standard deviation
SE: Standard error
