# Supplementary material for: Association of Single Measurement of dipstick proteinuria with physical performance of military males: the CHIEF study
Source: BMC Nephrol. 2020 Jul 18;21:287. doi: 10.1186/s12882-020-01948-w (PMC7368697; doi:10.1186/s12882-020-01948-w)
Supplement: Supplementary file 1 — Additional file 1. [file 12882_2020_1948_MOESM1_ESM.docx]

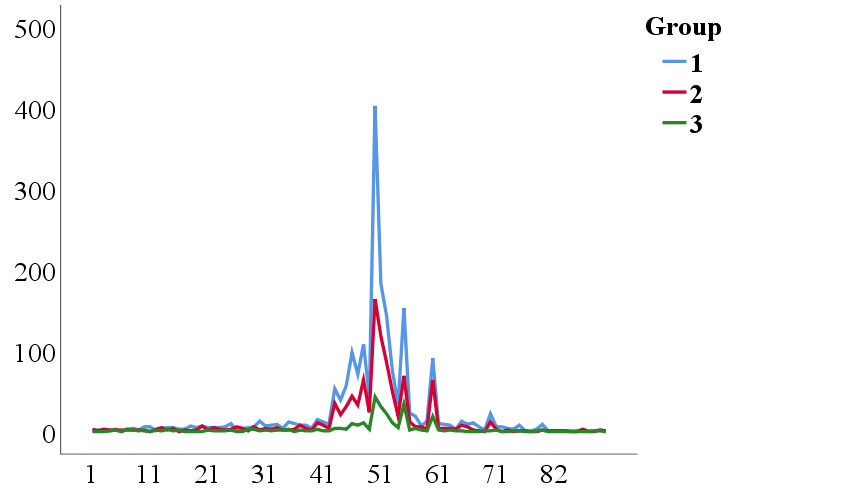

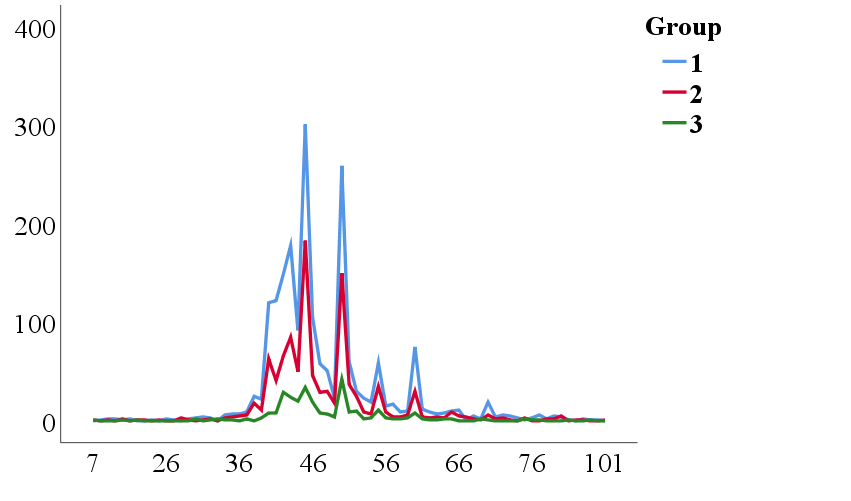


**Males (numbers)**

**Males (numbers)**

**2-min sit-ups (numbers)**

**2-min push-ups (numbers)**


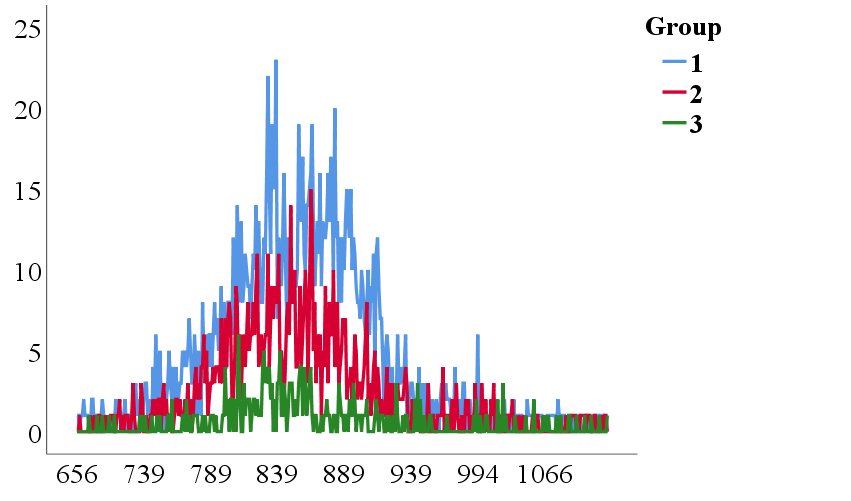


**Males (numbers)**

**3000-m run (seconds)**

Group 1 is denoted as participants with unremarkable proteinuria.

Group 2 is denoted as participants with moderate proteinuria.

Group 3 is denoted as participants with severe proteinuria.
