# Supplementary material for: Association of Single Measurement of dipstick proteinuria with physical performance of military males: the CHIEF study
Source: BMC Nephrol. 2020 Jul 18;21:287. doi: 10.1186/s12882-020-01948-w (PMC7368697; doi:10.1186/s12882-020-01948-w)
Supplement: Supplementary file 2 — Additional file 2. [file 12882_2020_1948_MOESM2_ESM.docx]

**Supplemental Table 1.** Differences in Each Exercise Performance in the Women-Only Cohort.

|  |  | 2-min push-ups (numbers) | | |  | 2-min sit-ups (numbers) | | |  | 3000-m run (seconds) | | |
| --- | --- | --- | --- | --- | --- | --- | --- | --- | --- | --- | --- | --- |
| Model 1 |  | n | Mean(SE) | *p*-value |  | n | Mean(SE) | *p*-value |  | n | Mean(SE) | *p*-value |
| Unremarkable proteinuria |  | 346 | 34.1 (0.5) | 0.16 |  | 362 | 37.7 (0.4) | 0.57 |  | 285 | 1005.8 (4.3) | 0.36 |
| Moderate or severe proteinuria |  | 45 | 31.9 (1.5) |  |  | 46 | 37.1 (1.1) |  |  | 35 | 1017.4 (12.2) |  |
| Model 2 |  |  |  |  |  |  |  |  |  |  |  |  |
| Unremarkable proteinuria |  | 344 | 34.2 (0.5) | 0.15 |  | 360 | 37.7 (0.4) | 0.56 |  | 283 | 1005.8 (4.0) | 0.44 |
| Moderate or severe proteinuria |  | 45 | 31.9 (1.5) |  |  | 46 | 37.0 (1.0) |  |  | 35 | 1015.2 (11.5) |  |
| Model 3 |  |  |  |  |  |  |  |  |  |  |  |  |
| Unremarkable proteinuria |  | 343 | 34.2 (0.5) | 0.10 |  | 359 | 37.7 (0.4) | 0.60 |  | 282 | 1005.8 (4.0) | 0.45 |
| Moderate or severe proteinuria |  | 45 | 31.7 (1.5) |  |  | 46 | 37.1 (1.0) |  |  | 35 | 1015.0 (11.4) |  |

Mean ± standard error (SE) for each exercise performance estimated using analysis of covariance with adjustments for

Model 1: age and service specialty adjustments;

Model 2: the covariates in Model 1, body mass index, BUN and serum creatinine adjustments;

Model 3: the covariates in Model 2, systolic blood pressure, fasting plasma glucose, alcohol intake status, cigarette smoking status, and weekly physical activity adjustments.

**Supplemental Table 2** Multiple Liner Regressions of Dipstick Proteinuria Severity With Each Exercise Performance in the Women-Only Cohort.

|  |  | Moderate to severe proteinuria | | |
| --- | --- | --- | --- | --- |
|  |  | β-value | 95% CI | *p*-value |
| Model 1 |  |  |  |  |
| 2-min push-ups |  | -2.310 | -5.462 – 0.841 | 0.15 |
| 2-min sit-ups |  | -0.635 | -2.872 – 1.602 | 0.57 |
| 3000-m run |  | 12.101 | -13.249 – 37.451 | 0.34 |
| Model 2 |  |  |  |  |
| 2-min push-ups |  | -2.249 | -5.326 – 0.828 | 0.15 |
| 2-min sit-ups |  | -0.650 | -2.832 – 1.532 | 0.55 |
| 3000-m run |  | 9.339 | -14.609 – 33.286 | 0.44 |
| Model 3 |  |  |  |  |
| 2-min push-ups |  | -2.520 | -5.593 – 0.553 | 0.10 |
| 2-min sit-ups |  | -0.575 | -2.755 – 1.605 | 0.60 |
| 3000-m run |  | 9.120 | -14.645 – 32.885 | 0.45 |

Data are presented as βand 95% confidence intervals (CI) using Pearson’s correlation coefficient for

Model 1: age and service specialty adjustments;

Model 2: the covariates in Model 1, body mass index, blood urea nitrogen and serum creatinine adjustments;

Model 3: the covariates in Model 2, systolic blood pressure, fasting plasma glucose, alcohol intake status, cigarette smoking status, and weekly physical activity adjustments.

**Supplemental Table 3.** Multiple Logistic Regressions of Dipstick Proteinuria Severity With the Best and the Worst in Each Exercise Performance at the Levels of 5%, 10%, and 16%, Respectively

|  | Moderate proteinuria | | |  | Severe proteinuria | | |  | Unremarkable proteinuria |
| --- | --- | --- | --- | --- | --- | --- | --- | --- | --- |
|  | OR | 95% CI | *p*-value |  | OR | 95% CI | *p*-value |  | Ref |
| **Top 10% of performance level** |  |  |  |  |  |  |  |  |  |
| 2-min push-ups ≥60 numbers | 0.94 | 0.74 – 1.19 | 0.58 |  | 1.03 | 0.69 – 1.52 | 0.90 |  | 1.00 |
| 2-min sit-ups ≥59 numbers | 0.82 | 0.63 – 1.06 | 0.12 |  | 0.78 | 0.50 – 1.21 | 0.26 |  | 1.00 |
| 3000-m running ≤783 seconds | 1.04 | 0.86 – 1.26 | 0.66 |  | 0.90 | 0.65 – 1.25 | 0.52 |  | 1.00 |
| **Top 5% of performance level** |  |  |  |  |  |  |  |  |  |
| 2-min push-ups ≥65 numbers | 0.74 | 0.52 – 1.05 | 0.09 |  | 0.83 | 0.61 – 1.14 | 0.25 |  | 1.00 |
| 2-min sit-ups ≥63 numbers | 0.96 | 0.68 – 1.35 | 0.80 |  | 0.93 | 0.69 – 1.24 | 0.61 |  | 1.00 |
| 3000-m running ≤757 seconds | 1.08 | 0.88 – 1.34 | 0.46 |  | 0.95 | 0.79 – 1.14 | 0.57 |  | 1.00 |
| **Top 16% of performance level** |  |  |  |  |  |  |  |  |  |
| 2-min push-ups ≥55 numbers | 0.84 | 0.69 – 1.01 | 0.06 |  | 1.07 | 0.92 – 1.25 | 0.36 |  | 1.00 |
| 2-min sit-ups ≥54 numbers | 0.83 | 0.67 – 1.02 | 0.08 |  | 0.91 | 0.76 – 1.09 | 0.29 |  | 1.00 |
| 3000-m running ≤800 seconds | 1.06 | 0.89 – 1.26 | 0.53 |  | 0.89 | 0.76 – 1.04 | 0.14 |  | 1.00 |
|  |  |  |  |  |  |  |  |  |  |
| **Bottom 10% of performance level** |  |  |  |  |  |  |  |  |  |
| 2-min push-ups ≤37 numbers | 1.16 | 0.90 – 1.49 | 0.25 |  | 1.77 | 1.23 – 2.56 | <0.01 |  | 1.00 |
| 2-min sit-ups ≤40 numbers | 1.21 | 0.93 – 1.57 | 0.16 |  | 0.83 | 0.49 – 1.41 | 0.48 |  | 1.00 |
| 3000-m running ≥934 seconds | 1.30 | 0.99 – 1.69 | 0.05 |  | 1.93 | 1.31 – 2.84 | <0.01 |  | 1.00 |
| **Bottom 5% of performance level** |  |  |  |  |  |  |  |  |  |
| 2-min push-ups ≤23 numbers | 1.35 | 0.97 – 1.88 | 0.07 |  | 1.34 | 1.05 – 1.71 | 0.02 |  | 1.00 |
| 2-min sit-ups ≤39 numbers | 1.33 | 0.95 – 1.86 | 0.10 |  | 1.05 | 0.77 – 1.44 | 0.75 |  | 1.00 |
| 3000-m running ≥992 seconds | 1.44 | 0.99 – 2.08 | 0.05 |  | 1.59 | 1.25 – 2.03 | <0.01 |  | 1.00 |
| **Bottom 16% of performance level** |  |  |  |  |  |  |  |  |  |
| 2-min push-ups ≤44 numbers | 1.25 | 1.02 – 1.53 | 0.03 |  | 1.25 | 1.06 – 1.48 | <0.01 |  | 1.00 |
| 2-min sit-ups ≤41 numbers | 1.01 | 0.80 – 1.27 | 0.92 |  | 0.81 | 0.64 – 1.02 | 0.07 |  | 1.00 |
| 3000-m running ≥911 seconds | 1.09 | 0.87 – 1.36 | 0.45 |  | 1.30 | 1.10 – 1.54 | <0.01 |  | 1.00 |

Data are presented as odds ratios (OR) and 95% CI (confidence intervals) using multiple logistic regression analysis for Model 3: age, service specialty, body mass index, blood urea nitrogen, serum creatinine, systolic blood pressure, fasting plasma glucose, habits of alcohol intake, cigarette smoking, and weekly exercise frequency adjustments.
